# Engineering a Microfluidic Platform to Cryopreserve Stem Cells: A DMSO‐Free Sustainable Approach

**DOI:** 10.1002/adhm.202401264

**Published:** 2024-08-17

**Authors:** Saman Modaresi, Settimio Pacelli, Aishik Chakraborty, Ali Coyle, Wei Luo, Irtisha Singh, Arghya Paul

**Affiliations:** ^1^ Department of Chemical and Petroleum Engineering Bioengineering Graduate Program School of Engineering The University of Kansas Lawrence KS 66045 USA; ^2^ Department of Biomedical Engineering Illinois Institute of Technology Chicago IL 60616 USA; ^3^ Department of Chemical and Biochemical Engineering The University of Western Ontario London ON N6A 5B9 Canada; ^4^ Collaborative Specialization in Musculoskeletal Health Research and Bone and Joint Institute The University of Western Ontario London ON N6A 5B9 Canada; ^5^ School of Biomedical Engineering The University of Western Ontario London ON N6A 5B9 Canada; ^6^ Department of Cell Biology and Genetics College of Medicine Texas A&M University Bryan TX 77807 USA; ^7^ Department of Biomedical Engineering College of Engineering Texas A&M University College Station TX 77843 USA; ^8^ Interdisciplinary Program in Genetics and Genomics Texas A&M University College Station TX 77840 USA; ^9^ Department of Chemistry The Center for Advanced Materials and Biomaterials Research The University of Western Ontario London ON N6A 5B9 Canada

**Keywords:** cryopreservation, DMSO‐free cell banking, mechanoporation, microfabricated chip, microfluidics, stem cell freezing, trehalose delivery

## Abstract

Human adipose‐derived stem cells (hASCs) are cryopreserved traditionally using dimethyl sulfoxide (DMSO) as the cryoprotectant agent. DMSO penetrates cell membranes and prevents cellular damage during cryopreservation. However, DMSO is not inert to cells, inducing cytotoxic effects by causing mitochondrial dysfunction, reduced cell proliferation, and impaired hASCs transplantation. Additionally, large‐scale production of DMSO and contamination can adversely impact the environment. A sustainable, green alternative to DMSO is trehalose, a natural disaccharide cryoprotectant agent that does not pose any risk of cytotoxicity. However, the cellular permeability of trehalose is less compared to DMSO. Here, a microfluidic chip is developed for the intracellular delivery of trehalose in hASCs. The chip is designed for mechanoporation, which creates transient pores in cell membranes by mechanical deformation. Mechanoporation allows the sparingly permeable trehalose to be internalized within the cell cytosol. The amount of trehalose delivered intracellularly is quantified and optimized based on cellular compatibility and functionality. Furthermore, whole‐transcriptome sequencing confirms that less than 1% of all target genes display at least a twofold change in expression when cells are passed through the chip compared to untreated cells. Overall, the results confirm the feasibility and effectiveness of using this microfluidic chip for DMSO‐free cryopreservation of hASCs.

## Graphical abstract

A microfluidic chip‐based technology for cryopreserving stem cells is presented. The chip facilitates the internalization of the otherwise impermeable cryoprotectant agent trehalose. This technology is proposed as a sustainable and greener alternative to DMSO‐based cryopreservation.

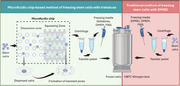

## 1 Introduction

Stem cells are a valuable resource for tissue engineering, regenerative medicine, and cell‐based therapies.^[^ ^1^, ^2^, ^3^, ^4^, ^5^, ^6^ ^]^ Currently, stem cells are being tested as a valid treatment approach in a variety of diseases such as leukemia, myocardial infarction, diabetes, autoimmune, and blood disorders.^[^ ^1^, ^7^, ^8^, ^9^ ^]^ Due to their immense potential in regenerative medicine, it is important to provide a safe method of cell storage post‐harvesting that can preserve their stemness and avoid metabolic damage during their cryopreservation. In addition, stem cell banking reduces the costs associated with culturing cells at 37 °C and limits the possibility of genetic alteration due to continuous long‐term culture in vitro.^[^ ^10^, ^11^, ^12^, ^13^ ^]^

Multiple strategies have been adopted for the cryopreservation of cells, including freeze‐drying,^[^ ^14^ ^]^ nanoparticle‐assisted freezing,^[^ ^15^ ^]^ biomimetic polymers,^[^ ^16^ ^]^ and liquid marble‐based microfluidic devices,^[^ ^17^ ^]^ among others. These techniques primarily aim at freezing without the need for cryoprotectant agents (CPAs). However, stem cell cryopreservation using different CPAs remains the most prevalent technique to date for cell freezing.^[^ ^18^, ^19^, ^20^, ^21^, ^22^ ^]^ The most common CPA is DMSO, a small amphiphilic molecule that rapidly penetrates the cell membrane and protects cells from irreversible damage during the freezing/thawing process.^[^ ^23^, ^24^ ^]^ Despite its efficiency, the use of DMSO for cryopreservation should be sparing. DMSO has been shown to be toxic to cells at 37 °C and requires rigorous washing steps for their complete removal from cells.^[^ ^25^ ^]^ Additionally, ≈10% of the cell population can be lost during each washing step, making this procedure highly inefficient.^[^ ^26^ ^]^ Further, DMSO is known to impair mitochondrial function and reduce cell proliferation.^[^ ^27^, ^28^ ^]^ Even at low concentrations, DMSO alters the epigenetic profile of cells, which leads to undesirable changes in cellular functions.^[^ ^29^ ^]^ DMSO has also been found to induce differentiation in more than 25 human stem cell lines,^[^ ^30^ ^]^ and incomplete removal of DMSO has been shown to cause intravascular hemolysis, increased serum transaminase levels, cardiac and pulmonary arrest, acute renal failure, and seizures.^[^ ^31^ ^]^ Additionally, DMSO production and contamination can have detrimental effects on the environment. It has been demonstrated that DMSO can adversely affect aquatic life.^[^ ^32^ ^]^ Also, DMSO contamination in soil can inhibit microbial oxidation of the greenhouse gas methane.^[^ ^33^ ^]^ Such impediments to natural methane removal from the environment can lead to potential global warming.

These concerning drawbacks make DMSO an unsustainable CPA, with limited clinical use.^[^ ^34^, ^35^, ^36^ ^]^ Subsequently, several alternatives to DMSO have been proposed for cryopreservation.^[^ ^37^, ^38^, ^39^ ^]^ Among them, safer alternatives in the form of disaccharides, such as trehalose, can be considered a natural CPA for cryopreservation.^[^ ^24^, ^40^, ^41^, ^42^, ^43^ ^]^ It offers the advantage of being nontoxic and does not need to be removed after thawing. However, one of the drawbacks associated with this type of CPA is the limited permeability across cell membranes.^[^ ^44^, ^45^ ^]^ To enhance permeability, mechanoporation can be introduced, which is a physical method of creating transient pores in cell membranes.^[^ ^46^ ^]^ Mechanoporation using engineered microfluidic chips is an emerging strategy for efficient intracellular delivery of exogenous bioorganic molecules.^[^ ^47^, ^48^ ^]^ Microfluidic chips with micron‐sized microfluidic constrictions of around half to one‐third of the cell's diameter can mechanically produce transient pores by cell squeezing.^[^ ^49^, ^50^, ^51^ ^]^ Additionally, a cell squeezing‐based strategy is cytocompatible, eliminates the need for chemical modification or toxic external delivery vehicles, and avoids harmful external energy sources for creating transient pores.^[^ ^52^, ^53^, ^54^ ^]^ Because of these inherent advantages, delivering exogenous bioorganic molecules to stem cells using microfluidic chip‐assisted mechanoporation has gained popularity recently compared to carrier‐based or chemical‐mediated delivery.

**Figure 1:**
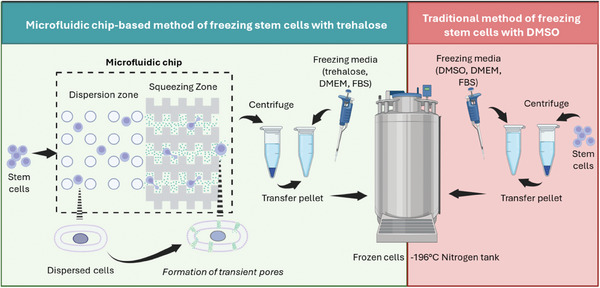
Microfluidic chip for trehalose‐based cryopreservation of cells as an alternative to traditional DMSO‐based approach. Illustration displays the method designed in our study. The microfluidic chip used here produces transient pores in hASCs by mechanoporation. These transient pores allow impermeable CPAs, such as trehalose, to go inside the cells. Subsequently, the cells can be cryopreserved in liquid nitrogen.

Here, we propose, for the first time, a sustainable green alternative to traditional DMSO‐based cryopreservation of hASCs using a microfluidic chip‐mediated strategy for delivering impermeable CPA trehalose intracellularly (**Figure** **1**). In this study, hASCs were selected because of their potential in regenerative medicine and cell‐based therapies. Moreover, polydimethylsiloxane (PDMS) used for fabricating the chip is a United States Food and Drug Administration (FDA) approved soft polymer that is well known for its biocompatibility.^[^ ^55^ ^]^ To validate the efficacy of our proposed method, we tested cell viability, apoptosis, stemness, angiogenic growth factor secretion, and differentiation capability post‐cryopreservation. hASCs frozen in freezing media containing DMSO were used as a control group for all the studies proposed in this work. Additionally, transcriptome‐wide sequencing was carried out to investigate the effect of cell squeezing‐based mechanoporation on the transcriptomic profile of the hASCs compared to untreated cells. Overall, the designed microfluidic chip can be a safe and environmentally sustainable alternative to DMSO‐based cryopreservation of hASCs.

## 2 Experimental Section

### 2.1 Materials

D‐(+)‐Trehalose dihydrate from Saccharomyces cerevisiae, phosphate buffer saline 1X (PBS), potassium phosphate monobasic (KH_2_PO_4_), potassium phosphate dibasic (K_2_HPO_4_), Magnesium chloride (MgCl_2_), DMSO, ascorbic acid‐2 phosphate, β‐glycerophosphate, dexamethasone, and hematoxylin were purchased from Sigma Aldrich. α‐Minimum Essential medium (α‐MEM), Dulbecco's Modified Eagle Medium (DMEM), fetal bovine serum (FBS), propidium iodide (PI), 10% goat serum, Mouse anti‐human Paxillin antibody, goat anti‐mouse AlexaFluor 594 antibody, diamidino‐2‐phenylindole dilactate (DAPI), phalloidin‐AlexaFluor488, Matrigel Matrix, and Gentamicin were purchased from Thermo Fisher Scientific. Trehalose fluorescein isothiocyanate (trehalose‐FITC) MW 731.7 Da was purchased from Kerafast.

### 2.2 Fabrication of Microfluidic Chip for the Delivery of Trehalose into hASCs

Silicone master molds needed for fabricating the PDMS microfluidic chips were prepared according to a protocol established previously.^[^ ^56^ ^]^ Briefly, a silicon wafer was exposed to hexamethyldisilazane (HMDS) at 150 °C for 30 min. Next, it was coated with a thin film of the negative photoresist, SU‐8 2015 (Microchem, Newton, MA). The wafer was then allowed to cool to room temperature. Next, a 15 µm thin layer of photoresist was added to the silicon wafer with a spin coater (3000 rpm, 30 s). The photoresist‐coated wafer was heated at 65 °C for 2 min and 95 °C for another 2 min to remove the solvent from the SU‐8 layer before UV crosslinking (Photronics Inc.). Two distinct chrome‐coated photomasks were then transferred onto the wafer with a contact‐type mask aligner (ABM Inc.). The photoresist was crosslinked by exposing UV light (365 nm) for 5–10 s, depending on the energy of the UV lamp. The UV‐crosslinked silicon wafers were heated at 95 °C for 10 min to complete the crosslinking procedure. Following the heating step, the wafer was washed with the SU‐8 developer solvent to remove any unreacted components. Finally, surface cracks were annealed by heating the wafer at 150 °C for 2 h. A brightfield microscope was used to confirm the geometry and size of the wafer. The thickness was measured using a profilometer. A PDMS prepolymer solution was used to fabricate the chip. However, prior to adding the prepolymer solution, the mold was treated for 30 min in a vacuum chamber with trimethylchlorosilane (TMCS). The TMCS treatment allowed an easy removal of PDMS post‐curing. The PDMS prepolymer solution was mixed with a curing agent (Sylgard 184 silicone elastomer kit, Dow Corning) at a ratio of 10:1. The resulting mixture was degassed, decanted onto the master mold, and cured for 3–4 h at 75 °C. After the curing process, the freshly formed microfluidic chip was removed from the mold, and holes (1 mm diameter) were punched to create the inlets and outlets of the chip. The PDMS was bound to the glass slides sealing the microfluidic channel using an oxygen plasma treatment for 40 s. To complete the binding process, the device was kept at 90 °C for 3 h. To complete the chip, Tygon tubes (1/16″ OD X 0.02″ ID) were placed inside the inlet and outlet holes using isopropanol. Microfluidic devices displaying a double deformation design with gaps of 8 µm in the deformation zones were selected for further studies.

### 2.3 Delivery of Trehalose into hASCs Using the Designed Microfluidic Chip

Human adipose‐derived stem cells, hASCs (passages 2–5) were used for all the studies. Cells were cultured in T‐75 flasks and collected by trypsinization when reaching 70–80% of confluency following the manufacturer's protocol. After the neutralization and removal of trypsin, the cell pellet was resuspended in α‐MEM and subdivided into aliquots containing 0.5 × 10^6^ cells. hASCs were centrifuged at a speed of 1.5 × 10^3^ g for 5 min. The media was discarded, and the pellet was resuspended in 0.5 mL of low‐conductive phosphate buffer (10 mm K_2_HPO_4_, 10 mm KH_2_PO_4_, and 1 mm MgCl_2_) containing different concentrations of trehalose (100, 200, 250, and 300 mm). Cell diameter was measured with an automated cell counter to evaluate the effect of the osmotic pressure of the delivery medium. Three measurements were carried out for each cell suspension. The microfluidic chip was washed and pretreated with the different trehalose solutions before the infusion of the cells. The cell suspension was then injected into the microfluidic chip at a constant flow rate of 150 µL min^−1^ using a microfluidic pump (Harvard Apparatus, MA, USA). hASCs were collected at the outlet and allowed to recover for 5 min post‐treatment at room temperature. Subsequently, the cell suspension was centrifuged at 1.5 × 10^3^ g for 5 min, and the supernatant containing trehalose was carefully removed and stored for further quantification, as reported in the following section. Cells were then resuspended in 0.5 mL of PBS 1X containing propidium iodide for 15 min at room temperature. Next, the cell suspension was analyzed with flow cytometry to evaluate the percentage of cell death.

### 2.4 Quantification and Visualization of Intracellular Trehalose

The amount of trehalose internalized by the cells was evaluated indirectly by measuring the concentration of trehalose before and after hASCs were squeezed in the microfluidic device. Precisely, trehalose was quantified by using a Megazyme trehalose assay^[^ ^57^ ^]^ (Megazyme International Ireland, Wicklow, Ireland), according to the instructions provided in the manufacturer's protocol. Trehalose is first enzymatically converted into glucose by hydrolysis. Glucose is then phosphorylated into glucose‐6‐phosphate (G6P) by a hexokinase. Finally, G6P is converted into gluconate‐6‐phosphate by the enzyme glucose‐6‐phosphate dehydrogenase (G6P‐DH) with the formation of reduced nicotinamide‐adenine dinucleotide phosphate (NADPH), which can be detected by UV spectroscopy at 340 nm using a well plate reader. The results were calculated as the mean ± standard deviation (*n* = 3).

Finally, to visualize the presence of intracellular trehalose, the same experiment was carried out by delivering trehalose‐FITC. Briefly, hASCs were suspended in a solution of trehalose‐FITC at the concentration of 0.125 mg mL^−1^ in PBS 1X. Cells were infused in the microfluidic chip and collected at the outlet. After squeezing, hASCs were left for 5 min at room temperature in the presence of trehalose‐FITC to allow the resealing of the membrane. hASCs were then centrifuged at the speed of 1.5 × 10^3^ g for 5 min, and the trehalose‐FITC solution was discarded. The pellet was resuspended in 0.35 mL of PBS 1X containing propidium iodide for 15 min at room temperature. Finally, 150 µL of a trypan blue 0.4% w/v solution was added to the cell suspension to quench the extracellular fluorescence of trehalose‐FITC. The efficiency of the delivery of trehalose‐FITC was evaluated by monitoring the percentage of fluorescent cells. The results were reported as the mean ± standard deviation (*n* = 3). Similarly, hASCs loaded with trehalose‐FITC were imaged using a fluorescent microscope (Evos Fl Auto, Life Sciences, USA), and corresponding brightfield images were taken to visualize the population of fluorescent cells.

### 2.5 Cryopreservation of hASCs Using Trehalose

hASCs (0.5 × 10^6^) were loaded with trehalose (300 mm) by following the procedure reported in Section 2.3. Cells were collected at the outlet of the microfluidic chip and allowed to reseal their membrane for 5 min at room temperature. Then, hASCs were centrifuged at 1.5 × 10^3^ g for 5 min, and the supernatant was discarded. The pellet was resuspended in freezing media containing trehalose as the CPA to obtain a cell suspension of 1 × 10^6^ cells mL^−1^. The freezing media was made of DMEM, 10% FBS, and trehalose included at different concentrations ranging from 50 to 600 mm. Cells were frozen at −80 °C for 24 h using a freezing container filled with isopropanol to allow the temperature to decrease constantly at the rate of 1 °C min^−1^. Subsequently, hASCs were stored in liquid nitrogen for 24 h. Additionally, hASCs were passed through the microfluid device without loading any trehalose intracellularly and frozen in the same freezing media containing trehalose. Finally, as a control group, hASCs cultured in flasks were collected by trypsinization and frozen at a cell density of 1 × 10^6^ mL^−1^ using DMEM, supplemented with 10% FBS and 10% DMSO. The cells were frozen following the same steps consisting of a slow freezing phase (1 °C min^−1^) at −80 °C for 24 h, followed by a freezing step in liquid nitrogen.

### 2.6 Assessment of Cell Viability, Morphology, Proliferation Rate, and Apoptosis Post‐Cryopreservation

The following studies were carried out using hASCs frozen in DMSO (control group) and hASCs loaded with trehalose (300 mm) and frozen in freezing media supplemented with trehalose (400 mm). The cryovials were removed from the liquid nitrogen tank and placed in a 37 °C beads bath for 5 min. The freezing media was diluted with an equal volume of fresh culture medium added to the cell suspension before centrifugation. The cells were resuspended in a PBS solution pH 7.4 supplemented with 1 µL of a PI solution (100 µg mL^−1^) for 15 min to assess cell viability. The percentage of dead cells post‐thawing was quantified with flow cytometry analysis by measuring the amount of red fluorescent cells. Three different samples were assessed for each tested group.

Cell viability, morphology, proliferation rate, and apoptosis were assessed using previously established protocols.^[^ ^56^ ^]^ hASCs obtained from the two different groups were thawed and seeded at a cell density of 1 × 10^4^ cells per well in a 24 well‐plate with flat and clear bottom. Cells were allowed to proliferate for 24 h before staining to visualize their morphology. Cells were fixed with 4% paraformaldehyde for 5 min at 37 °C and permeabilized with 0.1% Triton‐X100 for 10 min at room temperature. Samples were blocked with 5% normal goat serum at room temperature for 45 min. Mouse anti‐human Paxillin antibody (1:400 with 1% goat serum) was added to hASCs and left overnight at 4 °C. Goat anti‐mouse AlexaFluor 594 (1:500 in 1% goat serum) was then added and left for 1 h at room temperature. DAPI and phalloidin‐AlexaFluor488 were used to counterstain nuclei and *F*‐actin, respectively. Fluorescent images were taken for each sample with a fluorescent microscope (Evos Fl Auto) at 40X magnification. Images were processed using ImageJ software (NIH, USA), which enabled the quantitation of the total area of adhesion sites.

Furthermore, the proliferation rate of hASCs was monitored for 24 and 48 h post‐thawing using an MTS assay (Promega, USA). hASCs were seeded in 24 well plates at a cell density of 25 × 10^3^ cells per well. Cells were incubated with an MTS reagent (20 µL for every 100 µL of media) following the manufacturer's instructions. The absorbance of the media supplemented with the reagent was monitored at 490 nm after 1 h and 30 min of incubation at 37 °C in the dark. The proliferation rate was calculated considering an average of three different samples for each group and normalizing the results to the number of cells quantified 24 h post‐thawing. A calibration curve ranging from 5 × 10^3^ to 1 × 10^5^ cells per well was created to accurately correlate the absorbance values with the number of cells.

Finally, the population of apoptotic cells was determined by Annexin A5 staining and subsequently assessed by flow cytometry. Briefly, cells were thawed and seeded in 12 well plates at a cell density of 1 × 10^5^ cells per well. Cells were allowed to proliferate for 24 h prior to the experiment. Briefly, hASCs were collected by trypsinization and resuspended in PBS at a cell density of 1 × 10^6^ cells mL^−1^. Cells were stained with Annexin A5 following the instructions provided in the manufacturer's protocol and analyzed by flow cytometry. The results were reported as the mean ± standard deviation of three samples for each group.

### 2.7 Analysis of Stemness and Proapoptotic Genes Post‐Cryopreservation

The expression of key stemness markers was monitored by qPCR analysis to investigate whether the stemness of hASCs was preserved after being stored in freezing media supplemented with trehalose. Similarly, the expression of proapoptotic markers was investigated post‐cryopreservation. Briefly, cells were seeded in a 12‐well plate for 48 h after thawing. Two groups were tested, including hASCs frozen in DMSO and hASCs loaded with trehalose and frozen in media containing the disaccharide. mRNA was extracted from the cells using an RNeasy Mini Kit (Qiagen) and quantified using a NanoDrop spectrophotometer. Next, the mRNA samples were converted to cDNA using the High‐Capacity cDNA Conversion Kit (Applied Biosystems, USA). Finally, gene expression was measured using a mixture of predesigned primers and the KiCqStart SYBR Green Master Mix. The expression of the following stemness markers was assessed, including Klf4, Nanog, and Sox2. Similarly, the following proapoptotic genes, such as BAD, BAX, BAK1, and BBC3 were analyzed. All the reactions were performed using a Mastercycler RealPlex. The fold expression levels were calculated using the relative ΔΔCt method (*n* = 4). GAPDH was considered as the housekeeping gene, and results were normalized based on the gene expression of hASCs cryopreserved in freezing media‐supplemented DMSO.

### 2.8 Quantification and Assessment of Angiogenic Growth Factor Activity by hASCs In Vitro Post‐Cryopreservation

hASCs from the different groups were thawed and cultured in Rooster Bio medium containing 2% FBS at 37 °C and 5% CO_2_ for 24 and 48 h. Cells were seeded in 24 well plates at the cell density of 5 × 10^4^ cells per well. The conditioned media was collected, and the concentration of vascular endothelial growth factor (VEGF) was quantified using an ELISA kit (R&D Systems) (*n* = 3) by following the manufacturer's protocol. A standard curve in the range from 33 pg mL^−1^ to 1 ng mL^−1^ was used to correlate the absorbance at 450 nm with the concentration of VEGF.

Additionally, the conditioned media obtained from the different groups were tested for their efficacy in promoting the formation of tubular‐like structures in vitro following established procedures.^[^ ^58^ ^]^ Human umbilical vein endothelial cells (HUVECs) were seeded on a growth factor‐depleted Matrigel based on the manufacturer's protocol. Briefly, 289 µL of growth factor‐depleted Matrigel Matrix (10 mg mL^−1^) was previously thawed overnight on ice and added to a 24 well plate. The plate was then incubated at 37 °C for 60 min to allow the gel to form. HUVECs (1.2 × 10^5^) were seeded on each gel. The media composition was varied according to the different groups. The negative control consisted of endothelial cell growth medium‐2 bullet kit (EGM‐2, Lonza) without VEGF and basic fibroblast growth factor (bFGF). As a positive control, cells were cultured on the Matrigel with a Lonza Media EG2‐bullet kit supplemented with all the angiogenic growth factors. As test groups, HUVECs were cultured in EGM‐2 without angiogenic growth factor but supplemented with the condition containing VEGF at the concentration of 0.1 ng mL^−1^. After 18 h, brightfield images of the endothelial networks were taken for each group, and the total number of nodes, meshes, and segments, as well as the total segment length and the number of isolated segments were determined using the Angiogenesis Analyzer in ImageJ. Results were obtained from the analysis of at least 15 images for each group.

### 2.9 Evaluation of the Differentiation Ability of hASCs Post‐Cryopreservation

The differentiation potential of hASCs were demonstrated following a standard protocol.^[^ ^59^ ^]^ These studies were carried out to compare the differentiation potential post‐cryopreservation in vitro of hASCs frozen in media containing DMSO or trehalose. After thawing, hASCs were seeded at a cell density of 2.5 × 10^4^ cells per well in DMEM supplemented with 10% FBS and 1% penicillin and streptomycin. When cells reached 80% confluency, the media was replaced with complete adipogenesis differentiation medium (1X StemPro Adipocyte Differentiation Basal Medium, 1X StemPro Adipocyte Supplement, and Gentamicin (10 mg mL^−1^) reagent). The media was changed every other day for a total of 10 days. As a negative control group, hASCs were cultured in DMEM containing 10% FBS and 1% penicillin‐streptomycin for the entire duration of the study. On the last day of differentiation, the media was removed, and cells were washed with PBS. Cells were fixed with 4% paraformaldehyde for 5 min and washed with PBS. Subsequently, cells were treated with isopropanol 60% for 5 min. The presence of intracellular lipid droplets, which is indicative of adipogenic differentiation, was assessed by Oil Red O (Sigma–Aldrich) staining. The Oil Red Stock Solution was obtained by dissolving 60 mg of Oil Red O in 20 mL of 100% isopropanol. The suspension was mixed and allowed to sit for 20 min. Then, the Oil Red O stock solution was diluted in distilled water by mixing three parts of this solution with two parts of water. After 10 min, the suspension was filtered with a 0.22 µm syringe filter and used within 15 min. Four hundred microliters of Oil Red O solution was added to each well for 20 min. Cells were imaged using a microscope with a color camera to visualize stained lipid droplets.

Additionally, gene expressions of several markers involved in the early adipogenesis phase were monitored by qPCR analysis following the same procedure reported previously. These genes include peroxisome proliferator‐activated receptor gamma‐2 (PPARγ−2), lipoprotein lipase (LPL), adipocyte protein 2 (aP2), and GAPDH as the housekeeping gene.

Osteogenic differentiation was also investigated. After thawing, hASCs were initially cultured as reported above, and the media was replaced with an osteogenic medium consisting of α‐MEM, 10% FBS, 1% penicillin‐streptomycin, 50 µm ascorbic acid‐2 phosphate, 10 mm β‐glycerophosphate, and 10 nm dexamethasone. The media was changed every other day for 14 days. Alkaline phosphatase (ALP) enzymatic activity was measured at day 7 following a fluorometric assay (Abcam, USA). To measure the ALP activity, cells were centrifuged at 1.5 × 10^3^ g for 5 min, and the supernatant was removed. Cells were homogenized in ALP buffer following the steps described in the protocol provided by the kit. Fluorescence was measured using a microplate reader at the excitation of 360 nm and an emission wavelength of 440 nm (*n* = 3).

On day 14, the media was removed, and the cells were washed with PBS. The cells were fixed with 4% paraformaldehyde for 5 min. After fixation, hASCs were washed twice with distilled water and were stained for intracellular calcium deposits with 2% Alizarin Red S solution for 2–3 min. After washing the excess stain, cells were imaged with a microscope equipped with a color camera.

### 2.10 RNA‐Sequencing and Transcriptomic Analysis

Cells were first passed through the microfluidic chip based on the protocol outlined in Section 2.3. Subsequently, RNA was extracted, and the corresponding RNA sequencing was performed using an established protocol.^[^ ^60^ ^]^ Briefly, a high‐output NovaSeq platform was used on RNA samples with a read length of 125 bases (Charlie Johnson, Genomics and Bioinformatics Service, Texas, A&M AgriLife Research, College Station, TX). Trimming and aligning of the reads to the human genome (hg38) was done using Spliced Transcripts Alignment to a Reference (STAR).^[^ ^61^ ^]^ Principal Component Analysis (PCA) analysis was performed on 50% most variable genes. Differentially expressed genes (DEGs) were identified using the Bioconductor package, DESeq2, as described in prior studies.^[^ ^60^, ^62^ ^]^ The key processes that were affected were identified via Gene Ontology (GO) enrichment analysis using the Database for Annotation, Visualization, and Integrated Discovery (DAVID, https://david.ncifcrf.gov/) platform. DAVID was also used for Kyoto Encyclopedia of Genes and Genomes (KEGG) pathway analysis. For GO enrichment, only genes with False discovery rate (FDR)‐adjusted *p* < 0.01 and a differential fold ratio of at least 2 were selected. *R* was used to generate the plots.

### 2.11 Statistical Analysis

Statistical analysis was carried out by performing a two‐way analysis of variance (ANOVA) followed by Tukey's multiple comparison test, which was used to determine the presence of significant differences among the groups. A t‐test was used to compare experiments between two experimental groups. All statistical analyses were carried out with Graph‐pad Prism Software 7. A p‐value less than 0.05 indicates statistical significance, which was displayed as ^*^ *p* < 0.05, ^**^ *p* < 0.01, and ^***^ *p* < 0.001.

## 3 Results and Discussion

### 3.1 The Designed Cytocompatible Microfluidic Chip Facilitates the Internalization of Impermeable Molecules by Cell Squeezing while Preserving Cellular Activity of Stimulated Cells

**Figure 2:**
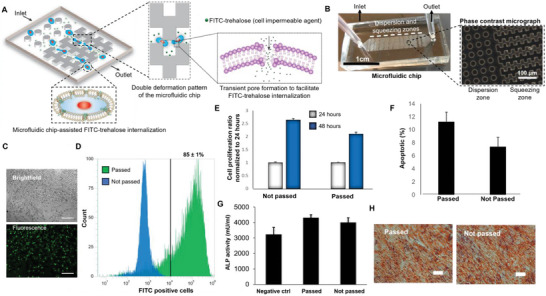
Microfluidic chip can create transient pores in hASCs to favor small molecule‐internalization. A) Schematic represents the process of cell squeezing necessary for the formation of transient pores in the cell membrane. These pores enable the passive diffusion of extracellular trehalose into the cells. B) Images display the fabricated microfluidic chip and corresponding phase contrast micrograph. Each chip is divided into 7 dispersion zones and 12 squeezing zones. The dispersion zone, which comprises of PDMS cylinders (20 µm diameter, 15 µm height, 30 µm gap), allows for the uniform distribution of the cells. The squeezing zones are placed in parallel and help in the steady flow of cells. C) Bright‐field image and corresponding fluorescent image of hASCs after delivery of insoluble trehalose‐FTIC molecules into the cells using chip‐based cell deformation method. Scale bars = 200 µm. Trehalose‐FITC has been used here as reporter molecules to easily visualize and quantify cell internalization. D) Quantification of intracellular delivery of trehalose‐FITC molecules into the hASCs (*n* = 3). Flow cytometry histograms of trehalose‐internalized hASCs after being passed through the microfluidic chip (passed) compared to trehalose‐FITC‐exposed untreated cells (not passed). The gate indicates the population of fluorescent cells that have internalized the disaccharide. E) MTS analysis of the cell proliferation rate of hASCs after passing through the microfluidic chip compared to untreated hASCs. Results were normalized based on the cell number obtained at 24 h. F) Quantification of apoptotic cells by flow cytometry analysis of hASCs after being passed through the microfluidic chip. The results were compared to untreated hASCs. Similar apoptotic percentage was observed between passed and not passed cells. G) Figure displays the bioactivity of the cells stimulated with the microfluidic chip. Alkaline phosphatase quantification of hASCs after 7 days of osteogenic differentiation has been shown here. The microfluidic chip did not affect the differentiating ability of hASCs. H) Images show alizarin red staining after 14 days of differentiation in osteogenic media. Scale bar = 200 µm. (*n* = 3). ^*^ = *p* < 0.05 ^**^ = *p* <0.01, and ^***^ *p* < 0.001.

Trehalose cannot diffuse through the cell membrane,^[^ ^41^ ^]^ and to overcome this issue, we have investigated a novel and safe delivery method that promotes effective internalization while retaining cell functionality. Toward this goal, hASCs were squeezed by passing them through micro‐constrictions in a microfluidic chip, which generated transient pores in the cell membrane (**Figure** **2**A,B). The microfluidic chip consists of two primary regions: a) dispersion zone and b) squeezing zone. The dispersion zone, with cylindrical patterns, disperses cells uniformly, whereas the squeezing zone comprises the micro‐constrictions necessary for cell squeezing. The resulting temporary defects in the cell membrane enable the passive diffusion of trehalose present in the delivery medium. Based on our previous work, a squeezing gap of 8 µm, a flow rate of 150 µL min^−1^, and a double deformation design were selected as the constant parameters of the microfluidic device in this study.^[^ ^56^ ^]^ This optimization was based on the internalization of a model macromolecule (dextran) as a function of the gap size, design type, and fluid flow rate.

To demonstrate the ability of our designed microfluidic chip to internalize impermeable molecules, we delivered trehalose‐FITC to hASCs and observed the fluorescence using optical microscopy. Video S1 (Supporting Information) displays the cell squeezing process using our designed microfluidic chip. Figure 2C shows a brightfield and corresponding fluorescent images of trehalose‐FITC internalized hASCs. Flow cytometry was then performed to quantify the percentage of cells that internalized the molecules (Figure 2D). Precisely, 85 ± 1% of the cell population was fluorescent after being squeezed through the microfluidic chip in the presence of trehalose‐FITC. Overall, these results indicate that our proposed strategy is efficient in promoting the delivery of trehalose. Next, we demonstrated that the designed microfluidic chip does not cause any significant cytotoxic or apoptotic effect (Figure 2E,F). Finally, we also verified whether the process of squeezing had any adverse effect on the differentiation potential of hASCs prior to freezing. The same studies have been carried out comparing the osteogenic and adipogenic differentiation of hASCs passed through the microfluidic device and untreated cells. hASCs passed through the microfluidic chip showed similar osteogenic behavior as untreated (not passed) cells, as shown by ALP quantification (Figure 2G) and Alizarin red staining (Figure 2H). Similarly, the process of squeezing did not alter the ability of the cells to differentiate toward the adipogenic lineage, and similar levels of staining and expression of LPL, aP2, and PPAR‐γ2 were found for both groups analyzed (Figure S1A,B, Supporting Information).

### 3.2 A Whole‐Transcriptome Analysis Validates Retention of Functionalities and Overall Cytocompatibility After Undergoing Microfluidic Chip‐Mediated Cell Squeezing

**Figure 3:**
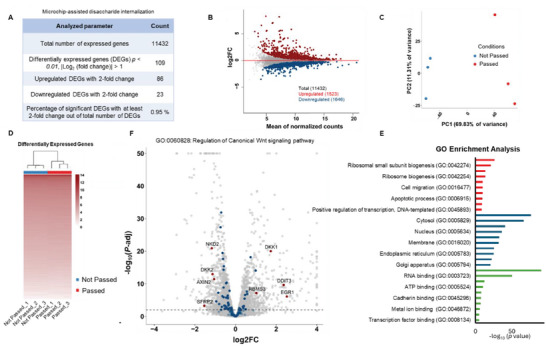
Transcriptomic profiles of hASCs using high throughput RNA‐seq demonstrate the feasibility of microfluidic chip for cryopreservation applications. A) Table displays the differentially expressed genes (DEGs) from RNA sequencing. Out of 11 432 expressed genes, only 109 shows more than two‐fold change in gene expression (*P*‐adj < 0.01). B) MA plot between the groups passed and not passed showing the upregulated (red) and downregulated (blue) DEGs (*P*‐adj < 0.01). Gray displays genes that did not show significant differences. C) Principal component analysis (PCA) of the samples based on mRNA expression obtained from RNA‐seq. The PCA was done on the mRNA expression (Log2FPKM) of 50% of the most variable genes. The data suggests the variation originates from the two groups and not the differences in the replicates. D) Hierarchical clustering of hASCs based on mRNA expression obtained from RNA‐seq. The heatmap shows FPKM of DEGs (*n* = 2286, *P*‐adj < 0.01) from cells passed through the microfluidic device compared to untreated cells (*n* = 3). E) Image illustrates GO functional analysis of DEGs (*P‐*adj *< 0.05*, |Log_2_ (fold change)| > 1) obtained using DAVID. The top 10 significant GO terms in GO categories of biological process, cellular component, and molecular function have been shown. GO enrichment analysis suggests that the passing of cells through the microfluidic chip is associated with various terms, including cell migration (Biological Process, GO:00 16477), membrane (Cellular Component, GO:00 16020), and cadherin binding (Molecular Function, GO:00 45296), among others. F) Volcano plot displaying the DEGs associated with canonical Wnt signaling pathway (GO:00 60828).

To further assess the cytocompatibility of cell squeezing using the designed microfluidic chip, we opted for a transcriptome‐wide approach characterizing the gene expression profile. In this regard, RNA‐seq is an effective tool for understanding the gene expression profile of cells.^[^ ^63^ ^]^ hASCs were first passed through the chip, and subsequently, RNA was extracted from the cells for further sequencing. Untreated hASCs that did not undergo microfluidic chip‐based mechanoporation were used as control (not passed). **Figure** **3**A shows the properties of the DEGs. Less than 1% of target genes significantly displayed at least a two‐fold change in the gene expression profile. Figure 3B displays an MA plot of the DEGs between the two groups (passed and not passed through the microfluidic chip). Principal component analysis (PCA) of mRNA expression levels (in FPKM – Fragments Per Kilobase of transcript per Million mapped reads) from RNA‐seq profiles of the experimental group and corresponding control has been shown in Figure 3C. Here, the PCA plot displays low variability in both groups (using 50% of most variable genes), suggesting the robustness of the samples studied. The heatmap shown in Figure 3D displays a similar expression profile among all DEGs between both groups and replicates (*N* = 2286 genes). To evaluate the impact of mechanoporation with the microfluidic chip on different biological pathways and processes, a gene ontology (GO) enrichment analysis was carried out next (Figure 3E). Passing cells through the microfluidic chip perturbed biological processes cell migration (GO: 00 16477, *p* < 1.47 × 10^−10^), negative regulation of cell proliferation (GO: 0 008285, *p* < 1.22 × 10^−10^), and cell cycle (GO: 0 007049, *p* < 5.21 × 10^−10^), suggesting cell migration and inhibition of proliferation after passing through the microfluidic chip. Based on cellular component, gene regulations were also associated with the expression of membrane‐specific genes, membrane (GO: 00 16020, *p* < 1.25 × 10^−32^), focal adhesion (GO: 0 005925, *p* < 1.6 × 10^−33^), and cytoplasm (GO: 0 005737, *p* < 1.56 × 10^−41^). Similarly, cell squeezing affected molecular functions enriching genes related to cadherin binding (GO: 00 45296, *p* < 3.33 × 10^−8^), collagen binding (GO: 0 005518, *p* < 8.75 × 10^−8^), and protein binding (GO: 0 005515, *p* < 2.48 × 10^−91^). Figure 3F highlights the volcano plot of the DEGs associated with the canonical Wingless and Int‐1 (cWnt) signaling pathway, GO: 00 60828. A limited number of genes from cWnt were significantly modulated, suggesting preservation of stemness (further supported by the JAK/STAT signaling pathway, Figure S2A, Supporting Information). Figure S2B,C (Supporting Information) shows minimal alterations in pathways related to mechanotransduction and apoptosis. Figure S3 (Supporting Information) displays the KEGG pathway enrichment analysis. The top 20 pathways with at least a two‐fold change in gene expression have been highlighted here.

### 3.3 Microfluidic Chip‐Mediated Trehalose Internalization Allows Successful Cryopreservation of hASCs

**Figure 4:**
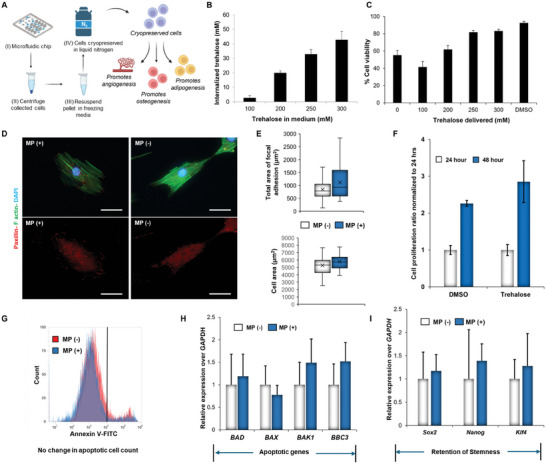
hASCs can be cryopreserved effectively by delivering trehalose with the developed microfluidic chip. A) The schematic shows the steps involved with cryopreservation using the designed microfluidic chip. After cryopreservation, the stem cells can be used for promoting angiogenesis, osteogenesis, and adipogenesis. B) Quantification of intracellular delivery of trehalose in hASCs (*n* = 3) has been displayed here. C) Cell viability of hASCs assessed by flow cytometry after cryopreservation. The amount of extracellular trehalose used in all the groups was 400 mm, except for DMSO. D) Retention of cell morphology and cytoskeletal organization. Images display immunofluorescence stained hASCs cryopreserved with freezing media supplemented with trehalose (400 mm) and DMSO (10% v/v). hASCs were loaded with trehalose intracellularly before cryopreservation by delivering 300 mm trehalose. Cells were stained with Alexa Fluor 488 Phalloidin to visualize actin stress fibers (green) and withDAPI to stain the nuclei (blue). In addition, paxillin staining (red) was carried out to identify the area of focal adhesions. Scale bar = 50 µm. E) Retention of cell adhesivity to substrates. The top graph quantifies the total area of focal adhesions expressed by hASCs cryopreserved with the two different types of freezing media. Results are calculated based on the red fluorescence intensity obtained from the immunofluorescent staining for paxillin expression (*n* = 20). The bottom graph quantifies the cell area of hASCs for the two groups tested. Results are reported as mean ± standard deviation (*n* = 17). F) hASCs undergoing cryopreservation using the two methods (traditional DMSO vs trehalose using microfluidic chip) did not show any significant differences in cell proliferation. MTS analysis of the cell proliferation rate of the two different groups was calculated at 48 h post‐thawing (*n* = 3). Results were normalized based on the cell number obtained at 24 h. G) The overlap between representative histogram plots obtained by flow cytometry displaying a similar population of apoptotic cells for both hASCs cryopreserved in freezing media containing DMSO and trehalose. The area in light blue defines the gate used to quantify the apoptotic cells stained with Annexin A5 (*n* = 3). H) qPCR analysis of key genes involved in the process of apoptosis such as *BAD*, *BAX*, *BAK1*, and *BBC3* 24 h post‐cryopreservation in DMSO and trehalose. I) Relative expression of stemness genes (*Sox2*, *Nanog*, *Klf4*) over *GAPDH* (housekeeping gene) in hASCs after 24 h post‐cryopreservation. qPCR results are reported as mean ± standard deviation (*n* = 3). ^*^ = *p* < 0.05, ^**^ = *p* <0.01, and ^***^ *p* < 0.001 (*n* = 3). MP (+) = hASCs cryopreserved using microfluidic chip supplemented with trehalose, MP (‐) = hASCs cryopreserved traditionally using DMSO.

**Figure** **4**A illustrates the devised cryopreservation strategy using the microfluidic chip‐assisted trehalose internalization. However, to be effective as a CPA, trehalose needs to be present both intracellularly and extracellularly.^[^ ^44^, ^64^ ^]^ Under dehydrated conditions, trehalose contributes to the formation of a stable glassy state that limits movement by establishing hydrogen bonds with proteins.^[^ ^65^ ^]^ Trehalose helps in maintaining protein structure and stabilizing phospholipid bilayers, which prevents cellular damage when freezing. Based on these premises, the first part of this study focused on optimizing the concentration of trehalose extracellularly and intracellularly in the freezing media to find an alternative to DMSO for stem cell cryopreservation. The amount of extracellular trehalose in the freezing media (DMEM containing 10% FBS) was optimized first based on the viability of hASCs. Specifically, the trehalose concentration varied from 50 to 600 mm to determine the optimal amount of extracellular trehalose needed for effective cryopreservation (Figure S4, Supporting Information). According to the results, 50 mm extracellular trehalose in the freezing medium showed a cell viability of 15 ± 3%. The viability gradually increased to 55 ± 8% with 400 mm of extracellular trehalose. However, a drop in viability was observed with higher concentrations. This drop was probably due to an increase in osmotic pressure, resulting in cellular damage. This osmotic effect was verified indirectly by measuring the variation in cell diameter of hASCs once resuspended in the freezing media containing trehalose prior to freezing. A decrease from 16.9 ± 0.2 to 14.02 ± 0.3 µm was measured as cells were exposed to freezing media supplemented with 50 and 600 mm trehalose, respectively (Figure S5A, Supporting Information). Therefore, 400 mm of extracellular trehalose was used for subsequent assays.

As the second step, we optimized the concentration of intracellular trehalose needed for cryopreservation. Trehalose was delivered intracellularly by varying its concentration from 100 to 300 mm using a low conductive phosphate buffer (≈50 mOsm kg^−1[^ ^66^ ^]^). The amount of trehalose delivered intracellularly was quantified indirectly by measuring the decrease in its concentration before and after squeezing the cells through the microfluidic device. As expected, the amount of disaccharide delivered intracellularly strictly depended on the initial concentration. The highest amount of trehalose delivered to the cells was 42.8 ± 5.76 mm, which was achieved by delivering trehalose at an initial concentration of 300 mm (Figure 4B). Aside from the concentration of trehalose, the cell diameter is another important parameter that can impact the squeezing process and the external cargo internalization. The best delivery results are commonly achieved when cells are squeezed within a gap with a size equal to half of the value of their diameter.^[^ ^67^ ^]^ The microfluidic chip used in this study possessed micro‐constrictions with a gap size of 8 ± 1 µm. This means that cells should display a diameter of 16 ± 1 µm to guarantee the maximum efficiency of delivery. The cell diameter was initially measured to assess any change induced by the different osmotic pressure of the trehalose solutions. Specifically, hypotonic (100 mm, ≈150 mOsm kg^−1^ and 200 mm ≈250 mOsm kg^−1^), isotonic (250 mm ≈290–300 mOsm kg^−1^) and hypertonic (300 mm, ≈350 mOsm kg^−1^) solutions were tested in this study. A constant decrease in cell diameter was observed from 21.7 ± 0.5 to 15.1 ± 0.2 µm as the concentration of trehalose was increased, suggesting a change in osmotic pressure (Figure S5B, Supporting Information). The low efficiency of internalization detected when using a solution of 100 mm of trehalose could be attributed to the largest cell diameter of hASCs in this delivery medium, which was equal to 21.7 ± 0.5 µm. This value is much higher than the optimal range of 16 ± 1 µm required to obtain a maximum delivery efficiency. On the contrary, the cell diameter was in the optimal range required for effective deformation using the designed microfluidic chip for concentrations of trehalose equal to 250 and 300 mm.

### 3.4 In Vitro Assays Substantiate the Cytocompatibility of Microfluidic Chip‐Mediated Cryopreservation of hASCs

hASCs loaded with trehalose were frozen in the freezing medium containing a constant concentration of extracellular trehalose of 400 mm, which was chosen based on our optimization process. Cell viability was assessed by flow cytometry analysis immediately after thawing in the media. Interestingly, the cell viability of hASCs after cryopreservation was significantly improved once trehalose was delivered intracellularly. Specifically, only 55.2% of hASCs were still alive after cryopreservation when no trehalose was internalized in the cytosol. As the concentration of trehalose delivered increased from 100 to 300 mm, a significant enhancement was detected with up to 83% cell viability (Figure 4C). However, there was an unexpected initial drop in cell viability, suggesting the requirement of a higher concentration of trehalose for cryopreservation. Hence, we tested cell viability with a higher concentration of trehalose in the freezing media. The morphology of the thawed hASCs was investigated next by immunofluorescence staining to visualize actin stress fibers and the expression of focal adhesion points. Specifically, staining for the presence of paxillin was performed, which is one of the main proteins present in the focal adhesion complexes of mammalian cells.^[^ ^68^ ^]^ Cells cryopreserved in trehalose (400 mm extracellular and 48.2 mm intracellularly) did not show any visible changes in morphology compared to the cells cryopreserved in the freezing media containing 10% of DMSO (Figure 4D). Quantification of the total area of focal adhesions and cell area showed comparable results for both tested groups (Figure 4E). Similarly, the proliferation rate of the cells post‐cryopreservation was monitored by MTS assays at 24 and 48 h. Cells were able to proliferate after 48 h with an increase in their cell number of 2.3 ± 0.1 and 2.86 ± 0.6 times for the DMSO and trehalose groups, respectively (Figure 4F). Furthermore, apoptotic cells were evaluated after 48 h post‐thawing by flow cytometry analysis. Specifically, cells were stained with Annexin A5, which is a protein that binds to phosphatidylserine.^[^ ^69^ ^]^ This phospholipid is normally present in the cytosol side of the plasma membrane in healthy cells.^[^ ^70^ ^]^ On the contrary, cells undergoing apoptosis start exposing this phospholipid on the extracellular side of their membrane, which can be detected by staining with Annexin A5 and quantified by flow cytometry. Similar percentages of apoptotic cells were found in both groups, with a value of 5.4 ± 0.3% and 6.2 ± 0.6% for trehalose and DMSO groups, respectively (Figure 4G).

### 3.5 Expression of Proapoptotic and Stemness Genes Remains Unaltered After Microfluidic Chip‐Mediated Cryopreservation of hASCs

The flow cytometry results were further substantiated by evaluating the expression of proapoptotic genes after 24 h post‐thawing. Precisely, we investigated the expression of several markers,^[^ ^71^, ^72^, ^73^ ^]^ such as BAD, BAX, BAK1, and BBC3, and the results were normalized based on the gene expression of hASCs frozen in DMSO. No significant difference was found in the expression of these markers for both groups investigated (Figure 4H). Finally, we evaluated the expression of key markers that regulate stem cells’ stemness,^[^ ^74^ ^]^ including Sox2, Nanog, and Klf4, after 24 h post‐thawing. Also, for this investigation, no significant difference in the expression of these genes was detected (Figure 4I). This data set demonstrated how hASCs cryopreserved with trehalose delivered intracellularly displayed a similar cellular behavior after thawing to the ones cryopreserved in DMSO. However, it should be noted that further investigation is required to verify whether similar results can be obtained for longer times of storage since data were collected after only 24 h post‐cryopreservation in liquid nitrogen.

### 3.6 Angiogenic Growth Factor Secretion and Proangiogenic Potential of the hASCs Were Sustained After Microchip‐Mediated Cryopreservation

**Figure 5:**
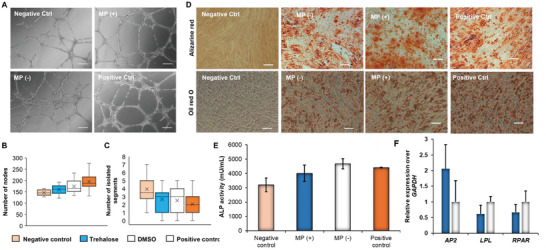
Microfluidic chip‐mediated cryopreservation retains the biological functionalities (such as angiogenesis and osteogenesis) of hASCs and their secretory profile. A) Retention of angiogenic potential of hASC‐secretome. The angiogenic potential of cell secretome secreted from cryopreserved hASCs has been tested here. Brightfield images display human endothelial cells cultured on Matrigel and treated with different types of secretome derived from hASCs cryopreserved in freezing media supplemented with trehalose, MP (+) or DMSO, MP (‐). The negative control group was represented by endothelial cells cultured without any angiogenic growth factor, whereas the positive control group was shown by endothelial cells cultured with angiogenic growth factors. Scale bars = 400 µm. Image J quantification of B) the number of nodes and C) the number of isolated segments has been graphed here. D) Retention of osteogenic potential of hASCs after cryopreservation. The ability of cryopreserved hASCs (trehalose vs DMSO) to differentiate under osteogenic and adipogenic media conditions has been assessed here. Alizarin red stain (top panel) to detect the presence of intracellular deposits of calcium in the different groups after 14 days demonstrates the ability of the cryopreserved hASCs to promote osteogenic differentiation. Oil Red O stain (bottom panel) of hASCs differentiated in the adipogenic medium after 10 days was performed to visualize the presence of intracellular lipid‐filled droplets. Additionally, the positive control refers to the cells grown in differentiation media. On the other hand, the negative control refers to the cells grown in regular serum‐supplemented growth media. The proposed method of cryopreservation did not affect the osteogenic and adipogenic differentiation ability of hASCs. Scale bars = 200 µm. E) Graph displays the alkaline phosphatase quantification of hASCs for the different groups after 7 days of osteogenic differentiation. F) qPCR analysis of *PPARγ−2*, *aP2*, and *LPL* genes, which are commonly upregulated during the process of adipogenic differentiation of stem cells, were performed after 10 days of differentiation. *GAPDH* was used as the housekeeping gene. Results are reported as mean ± deviation standard (*n* = 3). ^*^ = *p*<0.05, ^**^ = *p* < 0.01, and ^***^ = *p* < 0.001.

One of the most well‐known features of hASCs is their ability to produce a milieu of angiogenic growth factors that are essential to regulate angiogenesis both in vitro and in vivo.^[^ ^75^, ^76^, ^77^ ^]^ Therefore, it was imperative to first verify whether hASCs angiogenic potential was still retained by quantifying the amount of VEGF secreted after being mechanically squeezed through the microfluidic chip (Figure S6A). The results were expressed as a fold increase at 48 h and normalized to the amount of growth factor retrieved after 24 h. The fold increase of VEGF found in both groups was similar, indicating that the process of squeezing used to deliver trehalose retained the angiogenic potential of hASCs. The angiogenic potential of the different secretome was further evaluated by observing their ability to promote the reorganization of human umbilical vein endothelial cells (HUVECs) into tubular structures (Figure S6B). Brightfield images in Figure S6B demonstrates that the angiogenic potential of the hASC‐secretome was maintained after passing them through the microfluidic chip. Finally, we investigated the angiogenic potential of hASC‐secretome after cryopreservation using the developed method. A similar HUVEC‐based assay was conducted to determine the bioactivity of the hASC‐secretome (**Figure** **5A**). Particularly, HUVECs were seeded on Matrigel hydrogels, and their network structure was monitored over the course of 18 h. The medium used to culture HUVECs was supplemented with the secretome derived from the different groups of hASCs cultured for 24 h post‐thawing. Here, MP (+) refers to the secretome from hASCs cryopreserved using the microfluidic chip containing trehalose. On the other hand, MP (−) refers to the secretome from hASCs cryopreserved using the traditional DMSO method. Brightfield images were taken to assess the morphology of HUVECs and verify the formation of a healthy branched network. A positive and negative control were included in the study consisting of HUVECs cultured in Lonza medium with and without angiogenic growth factors, respectively. The morphology of the HUVECs’ network did not display significant differences among the groups except for the negative control, where a poor connection and lack of interconnected segments were evident after 18 h (**Figure** **5**A).

A more detailed analysis was carried out by ImageJ to quantify several parameters such as the number of nodes (Figure 5B), number of meshes (Figure S7A, Supporting Information), number of segments (Figure S7B, Supporting Information), the total segment length (Figure S7C, Supporting Information), and the number of isolated segments (Figure 5C). Each of these parameters is essential to define the connectivity of the network and the presence of tubular structures (meshes), which are indicative of a healthy vasculature network.^[^ ^78^ ^]^ On the contrary, the number of isolated segments should be as low as possible as it suggests the lack of connection among the branches of the network.^[^ ^79^ ^]^ As expected, the negative control displayed lower values among all groups for all the parameters quantified except for the total number of isolated segments. No significant difference was detected between the trehalose and the DMSO groups except for the total segment length, which was found to be significantly higher in the DMSO group. Overall, results suggest that the angiogenic secretome derived from hASCs cryopreserved with trehalose has a similar angiogenic potential with respect to hASCs cryopreserved in DMSO.

### 3.7 The Osteogenic and Adipogenic Differentiation Potential Was Retained by hASCs Cryopreserved Using the Designed Microfluidic Chip

The final step of this investigation aimed to verify the differentiation potential of hASCs into osteogenic and adipogenic lineages in vitro post‐cryopreservation with trehalose. A preliminary assay was done to demonstrate that the microfluidic chip retains the bioactivity of the hASCs passing through. Figure S8 (Supporting Information) shows no apparent changes in the osteogenic potential of hASCS after being passed through the microfluidic chip. The ALP activity of the trehalose group was comparable to that of DMSO. A qualitative evaluation of osteogenic differentiation has been shown by alizarin red staining (Figure 5D top panel). hASCs started to deposit a significant amount of intracellular calcium that was detected by alizarin red staining after 14 days post‐differentiation. No difference in the amount of intracellular calcium deposits was visible in the stained images of the cells cryopreserved with DMSO and trehalose after 14 days. Similarly, hASCs adipogenic differentiation was evaluated by staining with Oil red O to visualize the presence of intracellular lipid‐filled droplets. Overall, the DMSO and the trehalose groups displayed a similar level of staining after 10 days of adipogenic differentiation (Figure 5D bottom panel). Subsequently, a quantitative evaluation of osteogenic differentiation was conducted by assessing the presence of the enzyme alkaline phosphatase (ALP), which is generally considered an early osteogenic marker of differentiation.^[^ ^80^ ^]^ The ALP activity measured after 7 days of osteogenic differentiation was found to be similar between the DMSO and the trehalose group (Figure 5E). Further, the adipogenic differentiation of hASCs after 10 days of culture in an adipogenic medium was quantified. qPCR analysis of main adipogenic gene markers,^[^ ^81^ ^]^ such as LPL, aP2, and PPARγ−2, which are commonly upregulated in adipocytes, were quantified and compared between the two groups tested. Although aP2 was found to be expressed more in hASCs cryopreserved in trehalose, the results were not statistically significant for any of the markers tested (Figure 5F).

The results reported in this investigation are confirmed by other reports where trehalose was delivered intracellularly through alternative delivery methods. For instance, Dovgan et al. promoted the internalization of trehalose by electroporation in hASCs and found that the stem cells were able to successfully differentiate similarly into osteogenic and adipogenic lineages.^[^ ^66^ ^]^ In another study by Rao et al., trehalose was delivered using chitosan nanoparticles into hASCs.^[^ ^26^ ^]^ After cryopreservation, hASCs displayed similar differentiation potential toward adipogenic, osteogenic, and chondrogenic lineages with respect to cells without undergoing cryopreservation. Overall, these recent studies, combined with the findings reported in this work, strongly support the use of microfluidic chip‐assisted trehalose delivery as a safe alternative to DMSO for the cryopreservation of hASCs.

## 4 Conclusions

Our findings showed that microfluidic chip‐based DMSO‐free cryopreservation system is a feasible method for long‐term storage of hASCs. The designed chip can intracellularly deliver the CPA, trehalose by cell squeezing. The amount of disaccharide required to obtain the highest cell viability post‐cryopreservation had been optimized both intracellularly and extracellularly. Precisely, an extracellular concentration of 400 mm and an intracellular amount of 42.8 ± 5.76 mm were required to achieve the best results in terms of cell viability. Cells cryopreserved in trehalose displayed a similar proliferation rate, negligible apoptosis, and retention of stemness compared to cells cryopreserved in DMSO. Additionally, the angiogenic potential of the secretome collected from hASCs cryopreserved using trehalose was comparable to the one obtained from cells frozen with DMSO. Finally, we showed that the osteogenic and adipogenic differentiation potential of hASCs in vitro were preserved after cryopreservation. Furthermore, a thorough transcriptome‐wide sequencing using RNA‐seq revealed minimal differences in gene expression when the cells were passed through the microfluidic chip compared to untreated cells. Taken together, the fabricated microfluidic chip for the delivery of trehalose offers a feasible DMSO‐free alternative for cryopreservation. Additionally, the designed microfluidic chip may be considered for internalizing other impermeable CPAs such as glucose, galactose, fructose, maltose, and lactose, among others.^[^ ^82^ ^]^ As the field of cell therapy and tissue engineering is growing rapidly, our method of cryopreservation may be tested on other clinically relevant therapeutic cell lines, such as for Chimeric antigen receptor (CAR) T‐cells, bone marrow‐derived mesenchymal stem cells, hematopoietic stem cells, and neural stem cells, among others to demonstrate the versatility of this innovative approach. Moreover, the current study was a pilot project to prove the efficacy of our design. Future studies should consider large‐scale cryopreservation of stem cells and test for their long‐term storage to display the commercial feasibility of the product. Overall, the engineered microfluidic chip allows the efficient internalization of impermeable CPAs inside hASCs, providing us with a safe technology for stem cell cryopreservation.

## Conflict of Interest

The authors declare no conflict of interest.

## Author Contributions

S.M, S.P., and A.C. contributed equally to this work. S.M. performed methodology, investigation, data curation, and writing‐original draft; S.P. performed methodology, investigation, data curation, and writing‐original draft; A.C. performed methodology, investigation, data curation, and writing‐original draft; A.C. performed methodology, data curation, writing‐review and editing; W.L. performed data curation, writing‐review and editing; I.S. performed methodology, investigation, data curation, writing‐review and editing; A.P. performed conceptualization, methodology, supervision, funding acquisition, writing‐review and editing.

## Supporting information

Supporting Information

Supplemental Video 1

## Data Availability

The data that support the findings of this study are available from the corresponding author upon reasonable request. Additionally, the RNA‐seq data have been deposited in the Gene Expression Omnibus (GEO) database (accession no. GSE274972).
