# Supplementary material for: Engineering a Microfluidic Platform to Cryopreserve Stem Cells: A DMSO‐Free Sustainable Approach
Source: Adv Healthc Mater. 2024 Aug 17;13(29):2401264. doi: 10.1002/adhm.202401264 (PMC11582517; doi:10.1002/adhm.202401264)
Supplement: Supplementary file 1 — Supporting Information [file ADHM-13-0-s002.pdf]

ADVANCED HEALTHCARE MATERIALS

Supporting Information

for *Adv. Healthcare Mater.*, DOI 10.1002/adhm.202401264

# Engineering a Microfluidic Platform to Cryopreserve Stem Cells: A DMSO-Free Sustainable Approach

*Saman Modaresi, Settimio Pacelli, Aishik Chakraborty, Ali Coyle, Wei Luo, Irtisha Singh and Arghya Paul**

**Supporting Information**

# Engineering a microfluidic platform to cryopreserve stem cells: A DMSO-free sustainable approach

Saman Modaresi[a†], Settimio Pacelli[b†], Aishik Chakraborty[c,d†], Ali Coyle[e], Wei Luo[c], Irtisha Singh[f, g, h], Arghya Paul[c,d,e,i]

a Department of Chemical and Petroleum Engineering, Bioengineering Graduate Program, School of Engineering, The University of Kansas, Lawrence, Kansas, USA 66045
b Department of Biomedical Engineering, Illinois Institute of Technology, Chicago, Illinois, USA 60616
c Department of Chemical and Biochemical Engineering, The University of Western Ontario, London, Ontario, Canada N6A 5B9
d Collaborative Specialization in Musculoskeletal Health Research and Bone and Joint Institute, The University of Western Ontario, London, ON N6A 5B9, Canada
e School of Biomedical Engineering, The University of Western Ontario, London, Ontario, Canada N6A 5B9
f Department of Cell Biology and Genetics, College of Medicine, Texas A&M University, Bryan, TX 77807, USA
g Department of Biomedical Engineering, College of Engineering, Texas A&M University, College Station, TX 77843, USA
h Interdisciplinary Program in Genetics and Genomics, Texas A&M University, College Station, TX 77840, USA
i Department of Chemistry, The Center for Advanced Materials and Biomaterials Research, The University of Western Ontario, London, Ontario, Canada N6A 5B9

† Equal contributions.

Email: arghya.paul@uwo.ca

**Keywords:** Microfabricated chip, stem cell freezing, cryopreservation, trehalose delivery, DMSO-free cell banking, microfluidics, mechanoporation

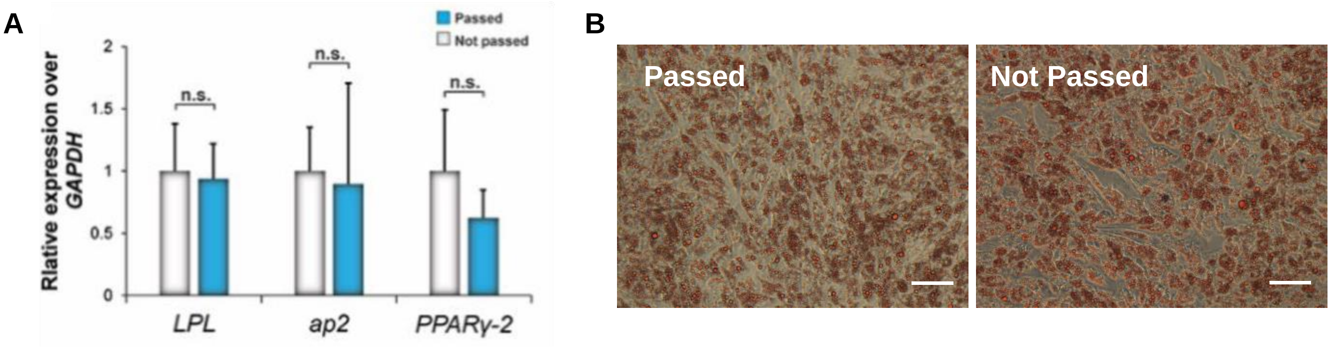


**Figure S1. Assessment of adipogenic differentiation of hASCs after being squeezed through the microfluidic chip.** (A) qPCR analysis of *PPARγ-2*, *aP2*, *LPL* genes, which are commonly upregulated during the process of adipogenic differentiation of stem cells after 10 days. (B) Oil red O stain showing the adipogenic differentiation of the different groups. Results are reported as mean ± deviation standard (n=3). * = p<0.05, ** = p<0.01 *** = p<0.001.

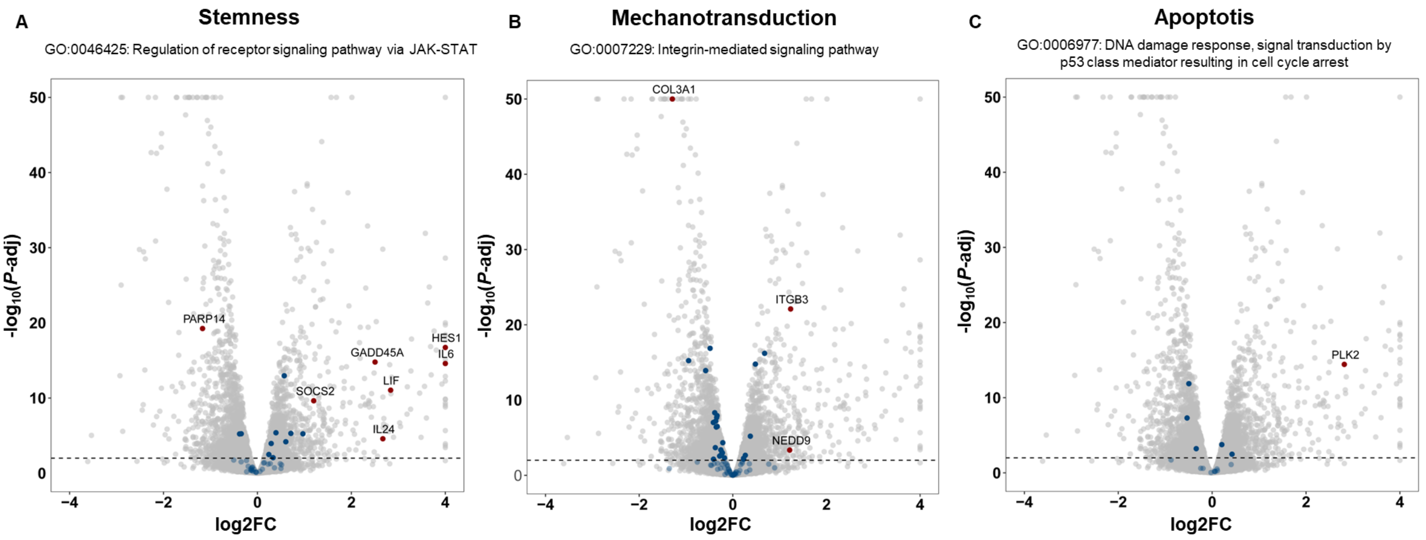


Significantly upregulated DEG: 13
Significantly downregulated DEG: 3

Significantly upregulated DEG: 7
Significantly downregulated DEG: 16

Significantly upregulated DEG: 3
Significantly downregulated DEG: 3

**Figure S2: Volcano plot of DEGs associated with (A) stemness, (B) mechanotransduction, and (c) apoptosis.** Very little changes in molecular pathways were observed when cells were passed through the designed microfluidic chip.

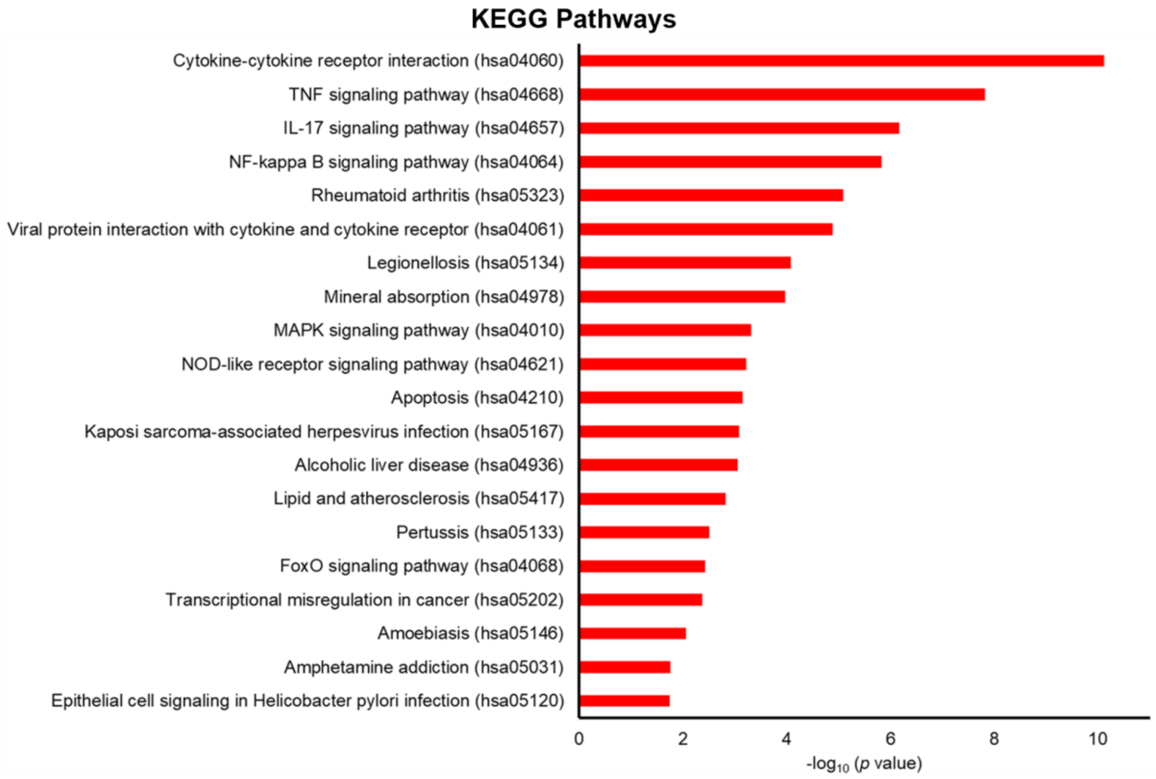


**Figure S3. RNA-seq analysis of cells passed through the microfluidic chip.** Graph highlights the Kyoto Encyclopedia of Genes and Genomes (KEGG) pathway enrichment analysis of the significant DEGs with at least 2-fold change in gene expression. The top 20 pathways have been shown here.

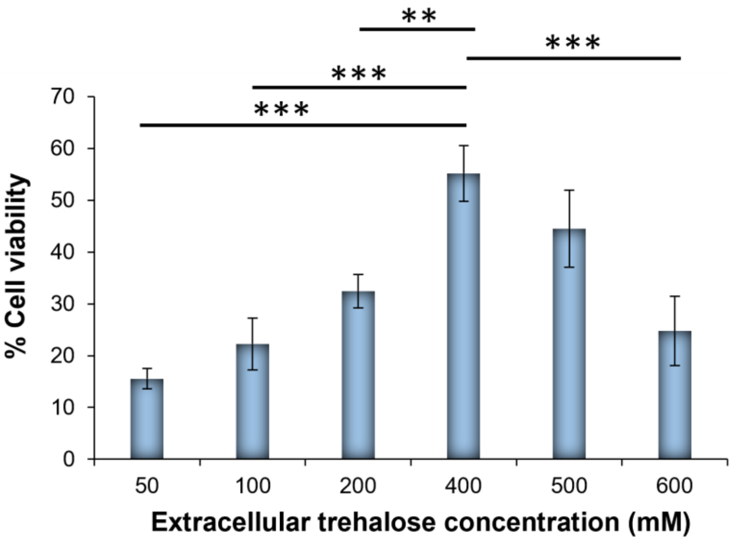


**Figure S4. Quantification of extracellular trehalose.** Post-thaw cell viability of hASCs assessed by flow cytometry to identify the optimal concentration of extracellular trehalose in the freezing medium.

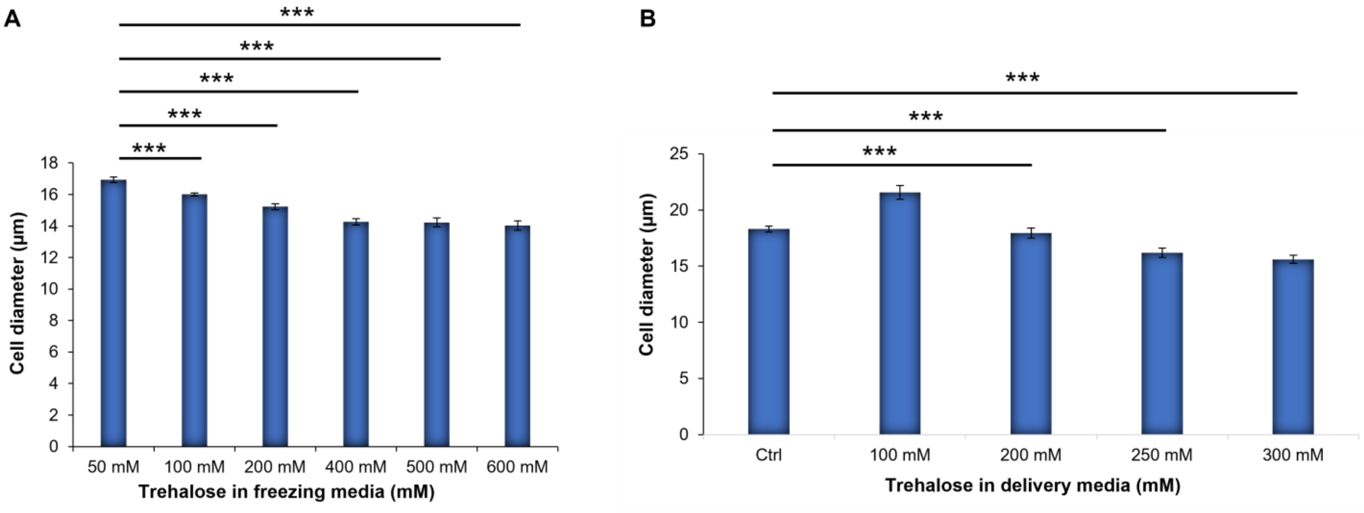


**Figure S5. Cell diameter measurements in different concentrations of trehalose.** (A) Cell diameter measurements of hASCs exposed to freezing media supplemented with different concentrations of trehalose ranging from 50 to 600 mM. (B) Cell diameter measurements of hASCs exposed to phosphate buffer (10 mM $K_2HPO_4$, 10 mM $KH_2PO_4$ and 1 mM $MgCl_2$) supplemented with different concentrations of trehalose varying from 0 to 300 mM. Results are reported as mean ± standard deviation (n=9). * = p < 0.05 ** = p <0.01, and *** p < 0.001.

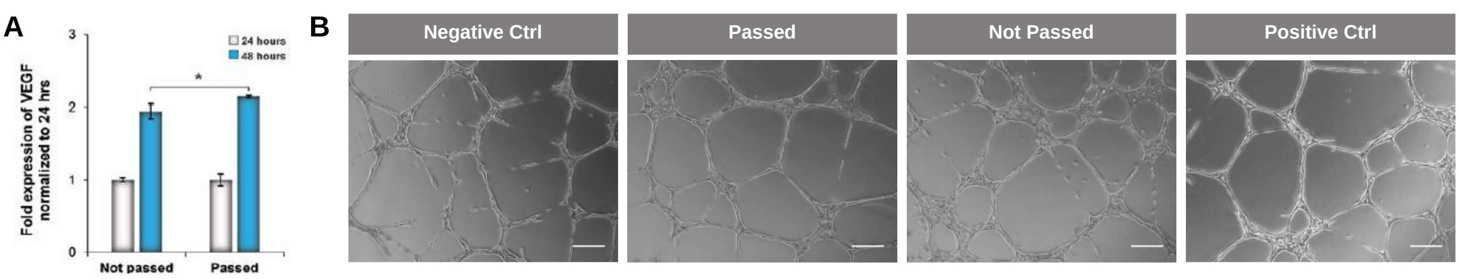


**Figure S6. Evaluation of the angiogenic potential of hASC (passed or not passed)-derived secretome tested on an *in vitro* HUVEC tube formation assay .** (A) Fold expression of VEGF secretion after 48 hours normalized to the amount of growth factor produced after 24 hours of culture. The two groups tested are hASCs passed and not passed through the microfluidic device. Results are reported as mean ± standard deviation (n=3). * = p < 0.05 ** = p <0.01, and *** p < 0.001. (B) Bright-field images of HUVECs cultured on Matrigel and treated with secretome derived from hASCs passed and not passed through the microfluidic device. The negative control group was represented by HUVECs cultured without any angiogenic growth factor, whereas the positive control group was represented by HUVECs cultured with angiogenic growth factors (manufacturer supplied). Scale bars = 400 µm.

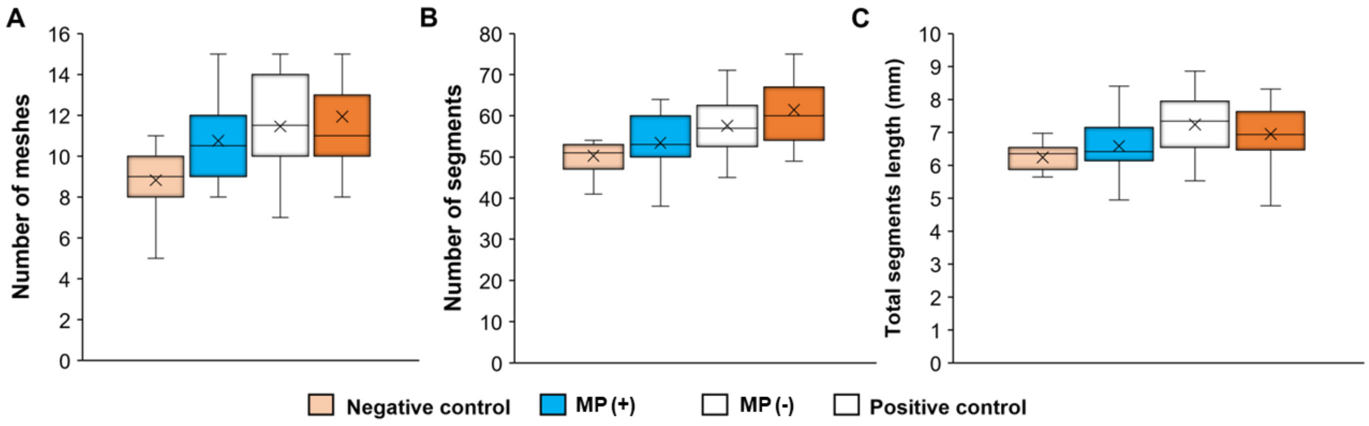


**Figure S7. ImageJ analysis of the bright-field images of HUVECs cultured on Matrigel.** Graphs display important factors considered for angiogenesis, including (A) number of meshes, (B) number of segments, and (C) total segments length.

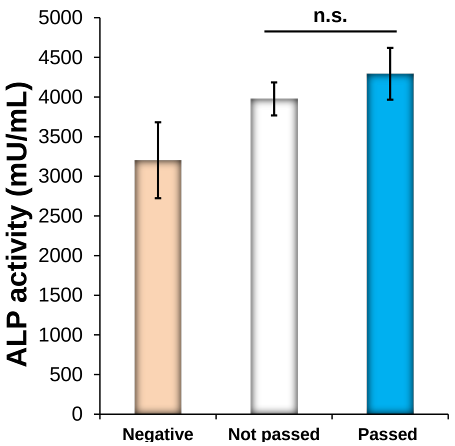


**Figure S8. Assessment of osteogenic differentiation.** Alkaline phosphatase quantification of hASCs for the different groups after 7 days of osteogenic differentiation. Scale bar = 200 µm. The results are reported as mean ± deviation standard (n=3). * = p<0.05, ** = p<0.01 *** = p<0.001, n.s. = not significant.
